# Posterior-only debridement, bone fusion, single-segment versus short-segment instrumentation for mono-segmental lumbar or lumbosacral pyogenic vertebral osteomyelitis: minimum five year follow-up outcomes

**DOI:** 10.1186/s13018-022-03269-0

**Published:** 2022-08-12

**Authors:** Hong-Qi Zhang, Yu-Xiang Wang, Chao-feng Guo, Ming-xing Tang, Shao-hua Liu, Ang Deng, Qile Gao

**Affiliations:** 1 Department of Spine Surgery and Orthopaedics, Xiangya Spinal Surgery Center, Xiangya Hospital of Central South University, Xiang Ya Road 87, Changsha, China; 2 National Clinical Research Center for Geriatric Disorder, Xiangya Hospital of Central South University, Changsha, China

**Keywords:** Mono-segmental, Lumbar, Lumbosacral, Pyogenic vertebral osteomyelitis, Titanium mesh cage, Posterior mono-segmental instrumentation, Posterior short-segmental instrumentation

## Abstract

**Background:**

Pyogenic vertebral osteomyelitis (PVO), which is a potentially life-threatening condition and is associated with significant morbidity and mortality, is a cause of back pain that can lead to neurologic deficits if not diagnosed in time and effectively treated. The objective of this study is to compare the efficacy of posterior single-segment and short-segment fixation combined with one-stage posterior debridement and fusion for the treatment of mono-segmental lumbar or lumbosacral PVO.

**Methods:**

Charts of all patients with mono-segmental lumbar or lumbosacral PVO were treated by single-stage posterior debridement, bone graft fusion, and pedicle screw fixation from April 2012 to January 2016. All patients were divided into two groups: sinlge-segment fixation (Group A, *n* = 31) and short-segment fixation (Group B, *n* = 36). These patients were followed up for a minimum of five years. The clinical efficacy was evaluated and compared on average operation time, blood loss, visual analog scale (VAS), erythrocyte sedimentation rate (ESR), C-Reactive protein (CRP), neurological function recovery and local lordotic angle.

**Results:**

All 67 patients were completely cured during the follow-up. All patients had significant improvement of neurological condition and pain relief at the final follow-up. The VAS was 7.1 ± 0.7 in group A and 7.2 ± 0.6 in group B pre-operatively, which decreased to 2.1 ± 0.6 and 2.0 ± 0.7, respectively, at three months after surgery, then reduced to 0.4 ± 0.5 and 0.5 ± 0.5, respectively, at the final follow-up. ESR, CRP returned to normal limits in all patients 3 months after surgery. The mean blood loss and operation time in group A were less than that in group B (*P* < 0.05). The local lordotic angle in group A was increased from preoperative − 1.7 ± 7.9° to postoperative 5.8 ± 7.1°, with angle loss of 1.5 ± 0.8° at the final follow-up, respectively (*P* < 0.05). The local lordotic angle in group B was increased from preoperative − 1.6 ± 7.8° to postoperative 13.5 ± 6.2°, with angle loss of 1.3 ± 0.8° at the final follow-up, respectively (*P* < 0.05). In the mean postoperative local lordotic angle, there was significant difference between the two groups at the time of immediate postoperative period or the final follow-up (*P* < 0.05).

**Conclusion:**

Posterior-only debridement, interbody graft using titanium mesh cage, posterior single-segment instrumentation and fusion represent a safe and effective treatment option for selected patients with mono-segmental lumbar and lumbosacral PVO. This approach may preserve more lumbar normal motor units with less blood loss and operation time when compared with that of short-segment fixation. But short-segment fixation was superior to the single-segment fixation in the correction of kyphosis.

## Introduction

Pyogenic vertebral osteomyelitis (PVO) comprises less than 4% of all bone infections [1], but can be associated with devastating morbidity and mortality, resulting in significant pain, deformity and neurological deficit [2]. The diagnosis and management of PVO remains difficult, not least because of the increasing elderly and immuno-compromised population worldwide. The majority of patients with PVO can be treated nonsurgically with appropriate antibiotics and external immobilization, particularly if the diagnosis is made early and the causative organism can be identified from a closed needle biopsy [3]. As such, indications for surgery have been limited to the unsuccessful conservative therapy, neurologic impairment, epidural abscess formation, intractable pain, or vertebral destruction leading to early or late spinal instability or segmental kyphosis [4, 5]. However, the current surgical treatment of PVO is still controversial, especially the choice of fixed segment length and selection of surgical approach. Although anterior approach have been conventionally preferred, complicated anatomic layers, segmental vessels, occasionally major vessels, and nerves should not be overlooked [6]. Furthermore, some patients often combined with cardiovascular and respiratory disease and single lung ventilation via anterior approach could result in more complications [7]. The combined posterior and anterior procedures lead to increased operating time, prolonged anesthesia, greater blood loss and increased mortality, morbidity and complications.

Posterior instrumentation has become popular as a technique for correction of kyphotic deformity and stabilization of the unstable spine in the past decade. In addition, we have applied posterior-only surgery in the treatment for spinal tuberculosis, which have got satisfactory clinical effects [7–9]. In view of this, posterior-only surgery was used in the treatment of mono-segmental lumbar or lumbosacral PVO.

There are no comparative studies on mono-segmental lumbar or lumbosacral PVO treated by posterior-only debridement, fusion, and single-segment vs. short-segment fixation. Therefore, the aim of our clinical study was to compare the clinical and radiological outcomes of posterior single-segment and short-segment fixation combined with posterior-only debridement and bone grafting fusion in treating mono-segmental lumbar or lumbosacral PVO.

## Material and methods

This study was a retrospective case series (level 4 evidence) and was approved by the ethics board committee of our hospital. Charts of all patients with mono-segmental lumbar or lumbosacral PVO treated from April 2012 to January 2016 by posterior-only debridement, interbody bone graft using titanium mesh cage, posterior instrumentation and fusion were retrospectively reviewed.

Patients whose major lesion involves mono-segmental with any of the following conditions were selected: (1) failure of conservative treatment, (2) intractable back pain, (3) vertebral destruction causing spinal instability, (4) neurological deterioration, (5) obvious abscess formation.

Exclusion criteria were the following: (1) multi-segmental involvement with severe destruction of vertebral bodies, (2) multi-level large paraspinal abscesses or gravitation abscess, (3) previous lumbar or lumbosacral surgery.

According to the length of fixation, the cases were divided into single-segment fixation group (group A, where the fixed/fused range was limited to only one damaged motion segment) and short-segment fixation group (group B, where the fixed/fused range included both the one damaged segment and one normal motion segment located above and below the damaged motion segment, respectively). In group A, there were 31 cases (19 males and 12 females) with a mean age 44.4 years (range 32 to 57 years). In group B, there were 36 cases (22 males and 14 females) with a mean age of 44.8 years (range 35 to 61 years).

**Fig. 1:**
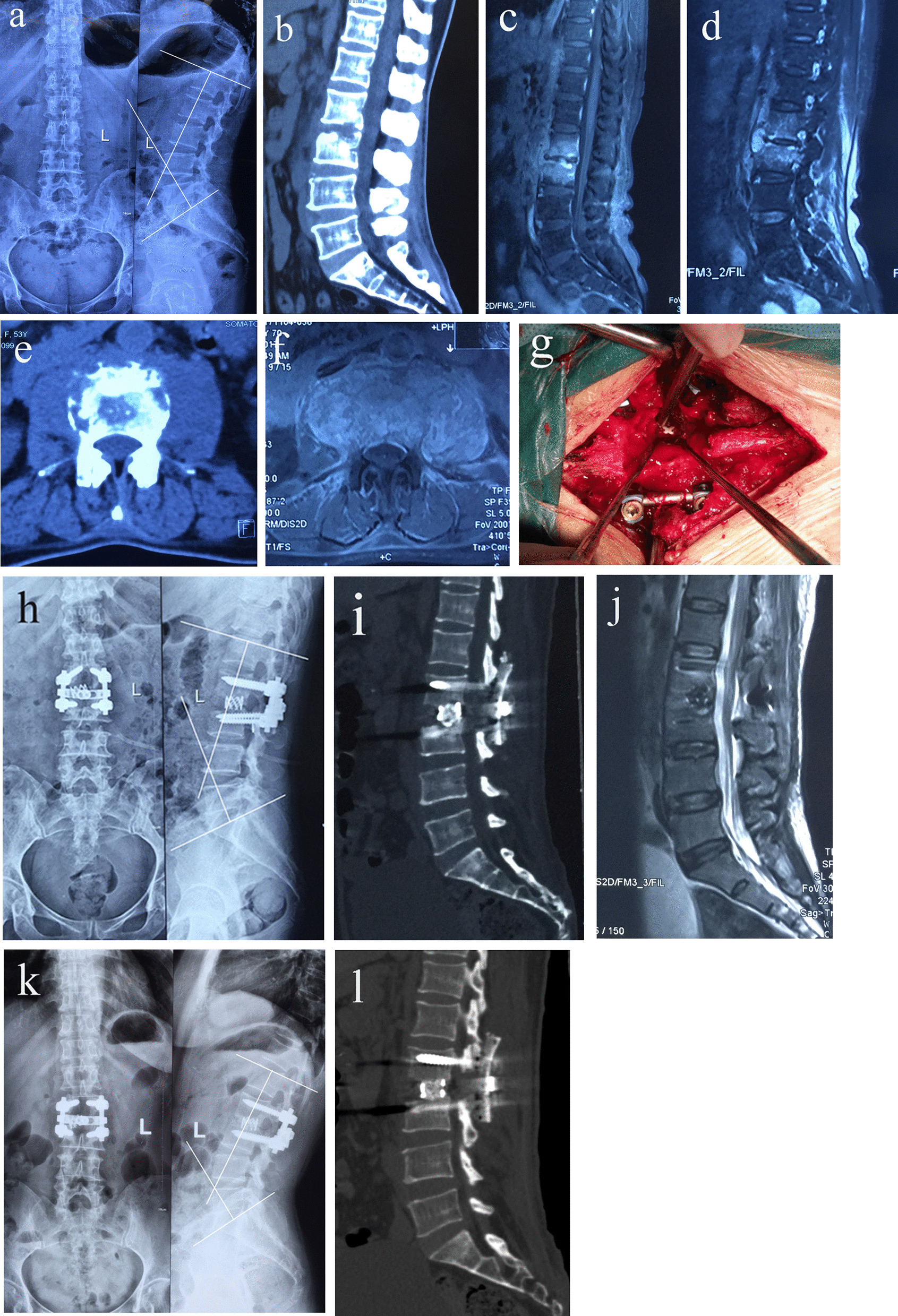
**a**–**f** Preoperative radiographs, CT and MRI showed that L2–L3 pyogenic vertebral osteomyelitis and lesion around vertebral body of L2–L3 developed paravertebral abscess with marked bony destruction. **g** Posterior interbody grafts using titanium mesh cages was applied posterolaterally. **h**–**j** Postoperative radiographs showed that posterior interbody graft using titanium mesh cage, posterior single-segmental instrumentation and fusion. Postoperative CT and MRI showed completely resolution of epidural abscess. Interbody graft using titanium mesh cages were placed satisfactorily. **k**, **l** Final follow-up radiographs and CT showed good bone fusion

**Fig. 2:**
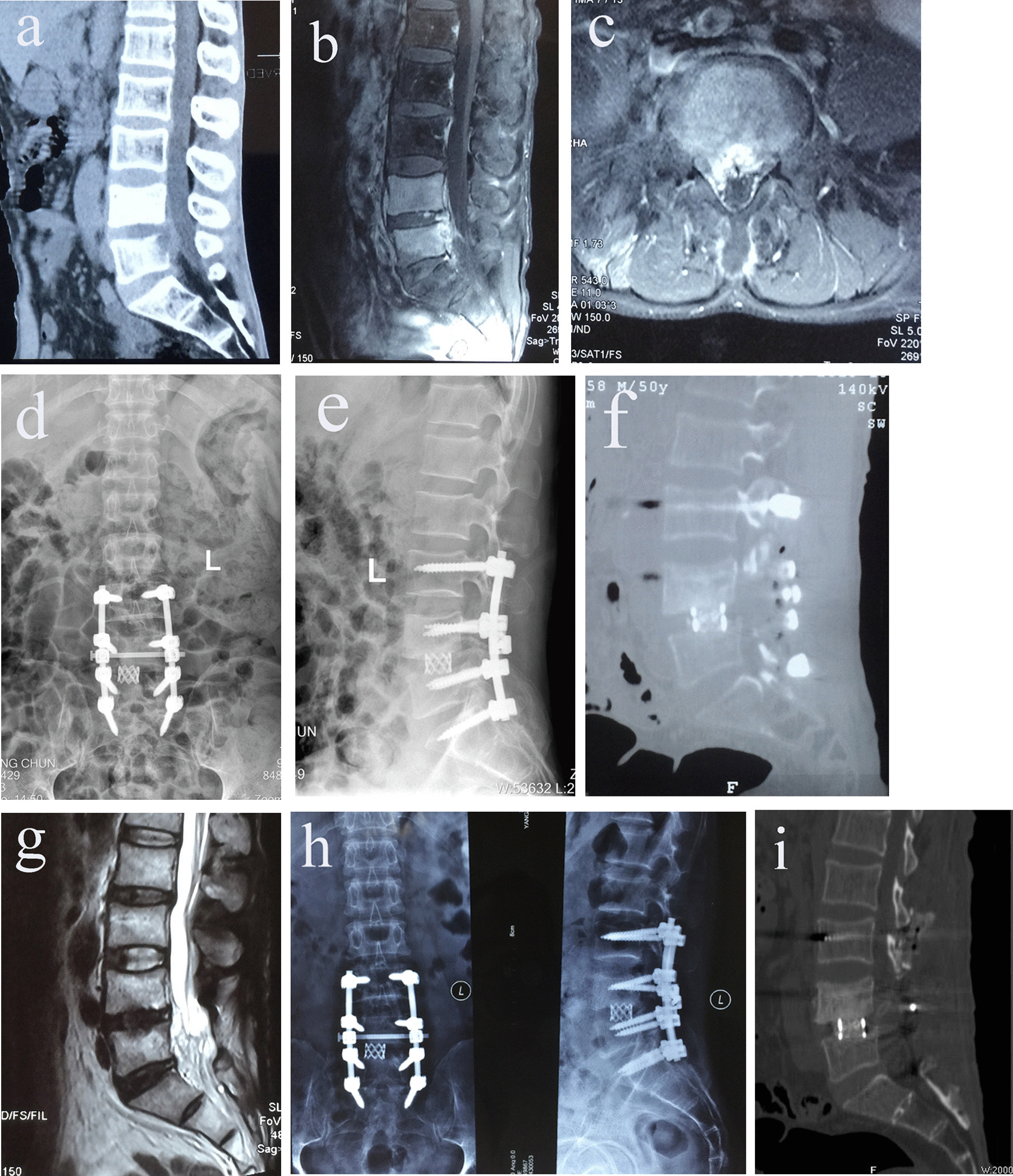
**a**–**c** Preoperative CT, and MRI showed that L4–L5 pyogenic vertebral osteomyelitis and lesion around vertebral body of L4–L5 developed paravertebral abscess with bony destruction.The abscess involved into the spinal canal with cord compromise resulted in neurologic deficit. **d**–**g** Postoperative radiographs and CT showed that interbody graft using titanium mesh cage, posterior short-segmental instrumentation and fusion. Postoperative MRI showed completely resolution of epidural abscess and decompression of neural component. **h**, **i** Final follow-up radiographs and CT showed good bone fusion

The main clinical symptoms of patients in two groups included fever, low back pain, lower extremity radiation pain, numbness, weakness, anorexia and weight loss. Hematological inflammatory indices included erythrocyte sedimentation rate (ESR), C-reactive protein (CRP), and total leukocyte count, which were documented preoperatively, postoperatively and at follow-up. Pain severity was assessed by visual analog scale (VAS). The American Spinal Injury Association (ASIA) was used to evaluate neurological function. All patients had imaging evaluations included radiographs, three dimensional computed tomography (CT), and magnetic resonance imaging (MRI) that were taken preoperatively to determine the affected level and detect vertebral body collapse, spinal instability, bone destruction, epidural and paravertebral abscess formation, and narrowing of the intervertebral space (Figs. 1a–f, 2a–c). The lumbar lordosis angle is the sagittal Cobb angle measured between the superior end plate of L1 and the superior end plate of S1 on the radiographs.

The diagnosis was based upon clinical presentation, imaging findings and laboratory examination, and subsequently confirmed by histopathological analysis of specimens obtained during surgery.

### Pre-operative management

Percutaneous biopsy of the affected vertebral bodies was performed on all patients posteriorly through a transpedicular approach under CT monitoring. All patients were in strict bed rest and were prescribed with continuous intravenous antibiotic therapy for at least 4 weeks before surgery except ten, of whom experienced neurological deterioration after 2–3 weeks of continuous intravenous antibiotic therapy.

### Surgical procedure

All surgical procedures were performed by the same group of surgeons. All patients were operated under general anesthesia in prone position.

In group A, through posterior midline approach, the lamina, facet joints, transverse processes were exposed. Exposing the vertebral laminae of involved segments, posterior pedicle screws were installed. Single segment fixation was performed. Transpedicular screws were only placed in the affected vertebrae. During transpedicular screw insertion, the site should be situated at a distance from the foci, but close to the end-plate to avoid exposing the screw after debridement. A temporary rod on the mild side of the focus was stabilized to avoid spinal cord injury induced by instability of the spine during decompression and focal debridement. After removing spinous process of the affected vertebrae, unilateral partial laminectomy or hemilaminectomy at the more severe lesion or more abscess side of the affected vertebrae was performed before debridement of the affected intervertebral discs and vertebrae. If necessary, unilateral partial or total facetectomy was also performed. Then, corpectomy and discectomy were performed, and abscess was evacuated. To achieve adequate debridement, compression wash and negative pressure suction were alternatively performed by inserting a urethral catheter into the abscess cavity. Spinal cord monitoring was also used, including motor-evoked and sensitive-evoked. One or two titanium mesh cages which were filled with allograft bone and autogenous bone, coming from healthy lamina, spinous process were shaped according to the shape and length of bone graft bed (Fig. 1g). Posterior interbody graft was applied posterolaterally. Interbody compression was performed after placement of interbody titanium mesh cages. Then, autogenous bone or an allograft of the proper size was selected for posterior fusion on the segments that underwent decompression and focal debridement. Tissue specimens obtained from the diseased spinal tissue at operation were sent for microbiological culture and histopathological analysis in all cases.

In group B, short segment fixation was performed, whose fixed range included both the one damaged segment and one normal motion segment located above and below the damaged motion segment, respectively. Other procedures were the same with group A.

### Post-operative management

Continuous flushing intervertebral space with 480,000 units of gentamicin and 3000 ml 0.9% sodium chloride solution was performed for 24 h after surgery in cases, which obvious purulent exudate was found intraoperatively. The drain was usually removed when irrigation of drainage fluid was negative for three times after surgery and white blood cell, ESR, CRP decreased obviousely earlier than before, which often lasted for 2–3 weeks. All patients were initially treated with intravenous broad-spectrum antibiotic, and then the antibiotic therapy was adjusted whenever organisms grown in cultures were identified and the sensitivities to antibiotics of these organisms were obtained. Intravenous antibiotic medications were continued for at least 4 weeks (range 4–6 weeks) after surgery or until all laboratory parameters in terms of white blood cell counts, ESR and CRP returned to normal limits. Patients were allowed to ambulate after operation for 14 to 20 days. The postoperative external support was needed for 3–6 months. Follow-up examination was performed during the first year at 4 weeks, 3, 6, 9 months and 1 year. Subsequent follow-ups were at yearly intervals. At each follow-up survey, they were assessed clinically for neurological function and pain and radiologically for spinal alignment and fusion progress. Plain radiographs were obtained at each follow-up measuring time to solid bony fusion (Figs. 1h, 2d, e). Successful fusion were defined as absence of local pain and tenderness over the site of fusion, no abnormal motion, no correction loss and hardware failure, presence of trabecular bone bridging between the grafts and the vertebrae, and no lucencies at the bone-cage interfaces. In inconclusive cases, bony fusion was assessed by three dimensional CT scan (Figs. 1i, j, 2f, g). Monitoring of laboratory parameters (white blood cell counts, ESR, CRP, hepatic function and renal function) was made regularly until these parameters returned to normal values. Complications were also recorded.

### Statistical analyses

SPSS24.0 statistical software was used for analysis.The clinical information between the two groups were compared using Student’ s *t* test and Wilcoxon signed-rank test. A rank sum test was used to analyze any discrepancy in normal data distributions. A *P*-value < 0.05 was considered statistically significant.

## Results

All 67 patients were completely cured during the follow-up. In group A, involved levels were observed at 5 cases in L1–2, 6 cases in L2–3, 6 cases in L3–4, 7 cases in L4–5, and 7 cases in L5–S1. In group B, involved levels were observed at 5 cases in L1–2, 6 cases in L2–3, 7 cases in L3–4, 10 cases in L4–5, and 8 cases in L5–S1.

Only 25 patients could be presumed for the source of the spinal infection despite careful diagnostic examination: 15 patients had undergone invasive procedures such as lumbar punctures and epidural injections before they experienced infection, of whom 9 patients were in group A, and 6 patients in group B. 4 patients had urinary tract infections, of whom 2 patients were in group A, and 2 patients in group B. In goup A, 2 patients suffered from bacteremia at another hospital, and a blood culture obtained postoperatively at that hospital revealed infection. 4 patients suffered from bacteremia at our hospital before surgery, and a blood culture obtained postoperatively at our hospital revealed infection, of whom one patient was in group A, 3 patients in group B. All patients had no recent (fewer than 12 months) nonspinal procedures or previous surgical interventions to the spine. Medical comorbidities in group A included diabetes mellitus in seven patients, smoking in seven patients, hypertension in three patients, chronic obstructive pulmonary disease (COPD) in two patients, hepatitis B in one patient and alcoholic hepatitis in two patients. Medical comorbidities in group B included diabetes mellitus in nine patients, smoking in nine patients, hypertension in four patients, COPD in one patient, hepatitis B in two patients.

All patients underwent blood culture and seven had urine culture done, but infectious organisms were isolated from blood culture in only ten patients, of whom one had positive urine cultures as well. The primary causative organism was *Staphylococcus aureus* in seven cases and *Escherichia coli* in three cases. Laboratory examination revealed a leukocytosis in 33 of the 67 patients. All patients had elevation of erythrocyte sedimentation rate (ESR) and C-reactive protein (CRP). In group A, the ESR ranged from 40 to 127 mm/h (average, 84.2 mm/h). The CRP ranged from 31 to 86 mg/L (average, 52.9 mg/L). In group B, the ESR ranged from 45 to 122 mm/h (average, 83.7 mm/h). The CRP ranged from 32 to 87 mg/L (average, 51.4 mg/L). Cultures of biopsy specimens were positive for *Staphylococcus aureus* in six patients, *Staphylococcus epidermidis* in two patients, *Escherichia coli* in two patients, but the diagnosis of PVO was confirmed histologically in all of them.

The mean periods of follow-up was 77.8 ± 10.5 months in group A and 80.9 ± 10.0 months in group B. The intra-operative blood loss and operation time in group B were more than that in group A, with a significant difference (*P* < 0.05) (Table 1). 54 patients of tissue specimens obtained from the diseased spinal tissue at operation had positive cultures. In group A, the most common causative organism was *Staphylococcus aureus*, which was positive in 10 patients. Other organisms were *Staphylococcus epidermidis* in 6 patients and *Pseudomonas* species in 3 patients; *Salmonella* species in 3 patients and *Escherichia coli* in 2 patients. In group B, the most common causative organism was also *Staphylococcus aureus*, which was positive in 12 patients. Other organisms were *Staphylococcus epidermidis* in 7 patients and *Salmonella* species in 4 patients; *Escherichia coli* in 4 patients and *Pseudomonas* species in 3 patients.

**Table 1:** The clinical data of patients

	Group A (*N* = 31)	Group B (*N* = 36)	*P* value
Gender (M/F)	19/12	22/14	0.99
Age (years)	44.4 ± 6.7	44.8 ± 6.3	0.69
Blood loss (ml)	447 ± 55	589 ± 86	< 0.01
Operation time (min)	177 ± 18	208 ± 17	< 0.01
Duration of follow-up (months)	77.8 ± 10.5	80.9 ± 10.0	0.22
Fusion time (months)	5.4 ± 1.0	5.2 ± 0.9	0.55
VAS			
Pre	7.1 ± 0.7	7.2 ± 0.6	0.27
Post	2.1 ± 0.6	2.0 ± 0.7	0.54
FFU	0.4 ± 0.5	0.5 ± 0.5	0.36
ESR(mm/h)			
Pre	84.2 ± 20.7	83.7 ± 17.2	0.92
TMP	9.0 ± 1.8	8.9 ± 1.5	0.49
CRP(mg/l)			
Pre	52.9 ± 14.7	51.4 ± 15.1	0.66
TMP	4.8 ± 1.2	5.0 ± 1.1	0.79

*M* male, *F* female, *Pre* pre-operative, *Post* post-operative, *TMP* three months post-operative, *FFU* final follow-up

Resolution of infection was exhibited in all, as noted by normalization of the ESR and CRP levels. All patients had significant improvement in constitutional symptoms and back pain after surgery. White blood cell, ESR, CRP returned to normal limits in all patients 3 months after surgery (Table 1). All patients had pain relief. The VAS was 7.1 ± 0.7 in group A and 7.2 ± 0.6 in group B pre-operatively, which decreased to 2.1 ± 0.6 and 2.0 ± 0.7, respectively, at three months after surgery, then reduced to 0.4 ± 0.5 and 0.5 ± 0.5, respectively, at the final follow-up (Table 1). All patients achieved bone fusion. The bony fusion time was 5.4 ± 1.0 months in group A and 5.2 ± 0.9 months in group B (Figs. 1k, l, 2h, i) (Table 1). No neurological deterioration after surgery was noted in any of the cases. Neurologic deficits improved at final follow-up (Table 2). At the last follow-up, the AISA grades of both two groups were significantly improved compared with that before operation (*P* < 0.05), and there was no significant difference between the two groups (*P* > 0.05) (Table 2). Mean preoperative local lordotic angle was similar between the two groups (Table 3). There was significant difference between the two groups at postoperative and final follow-up local lordotic angle (*P* < 0.05) (Table 3), and the local lordotic angle of the two groups was significantly improved compared with that before operation (*P* < 0.05) (Table 3). There was no obvious loss of correction in both groups (Table 3).

**Table 2:** Neurological status evaluated by the ASIA impairment scale

ASIA scale	Group A (*N* = 31)		Group B (*N* = 36)	
	Pre	FFU	Pre	FFU
A	0	0	0	0
B	1	0	2	0
C	4	0	3	0
D	12	2	14	3
E	14	29	17	33

*Pre* pre-operative, *FFU* final follow-up

**Table 3:** Comparison of the local lordotic angle

Group	*n*	Local lordotic angle (°)			Correction loss (°)
		Pre	Post	FFU	
A	31	− 1.7 ± 7.9#	5.8 ± 7.1*	4.3 ± 6.9*	1.5 ± 0.8
B	36	− 1.6 ± 7.8#	13.5 ± 6.2*	12.3 ± 6.0*	1.3 ± 0.8
Statistic		*t* = − 0.047	*t* = 4.075	*t* = − 5.125	*t* = 1.149
		*P* = 0.963	*P* = 0.000	*P* = 0.000	*P* = 0.255

*Pre* pre-operative, post post-operative immediately, *FFU* final follow-up

^*^Compared with pre-operative value, *P* < 0.05

^#^Compared with the value at immediately after operative, *P* < 0.05

### Complication

Postoperative skin infection occurred in two cases (1 in group A and 1 in group B), which were all cured after antibiotic therapy and wound dressing. No complications related to instrumentation occurred. Four patients (2 in group A and 2 in group B) suffered from pneumonia, resolutive with anti-inflammatory and symptomatic supportive treatment during one week. No graft fracture, sliding or resorption was observed.

## Discussion

Our study showed that all the patients in the two groups were cured without relapse, reinfection or long-term pain after surgery. All patients got bone fusion at the final follow-up without complications related to the internal fixation. This observation might indicate that both short-segment fixation and single-segment fixation can achieve the goal of long-term clinical treatment. And single-segment fixation techinique showed less invasive, which also can meets the requirements of reconstruction stability in the treatment of PVO. Single-segment fixation creates a relatively smaller surgical field of exposure and the procedure was performed with limited surgical access, thus causing little damage to the structure and physiological function of the spine, which is therefore less invasive, with reduced operative time and blood loss. Furthermore, in our study, the operation time and intra-operative blood loss in the single-segment fixation group were significantly lower than those in the short-segment fixation group, which brought relatively smaller surgical trauma.

From clinical perspective, previous studies have shown that the monosegmental fixation is a safe and effective technique in patients with spinal fractures with a minimum two years follow-up [10]. It can save motion segments in patients with adequate spine stability and good functional outcomes [10, 11]. Biomechanical experiments and finite element analysis have also indicated that single-segment fixation can meet the stability requirements necessary for spinal fracture reconstruction [12]. Single-segment fixation is more hard in the treatment of PVO than that in the treatment of spinal fracture due to the pathological features of PVO. The vertebrae infected by PVO often present new bone formation and bone sclerosis, so the bone mineral density of infected vertebrae is generally higher than normal vertebrae. This results in stronger holding forces in the vertebrae with infection lesions than in those vertebrae with fractures, when performing pedicle scews of placement. In our study, pedicle screws were often inserted close to the endplate, which could get stronger holding forces on the vertebrae. This can achieve good reconstruction of spinal stability, which can get pain relief. In our study, all patients experienced significant relief of low back pain after surgery.

The incidence of PVO is increasing, which may be attributable to various factors such as the aging of the society, the abuse of intravenous drugs, the widespread use of immunosuppression therapy for organ implant recipient and the progress in diagnostic methods with higher specificity [13–15]. These diseases often affect the “at-risk” populations, namely the elderly and the immunocomprimised due to malnutrition, diabetes mellitus, chronic smoking, intravenous drug abuse [13–15]. The most common site of infection is the lumbar spine (45–50%) [16]. Spinal infections are often preceded by infections elsewhere in the body; and predisposing conditions include a genitourinary infection, urinary tract intervention, intravenous drug abuse, immunosuppression, indwelling vascular catheter, diabetes mellitus [17, 18].

Despite recent advances in imaging, and microbiological and histopathological techniques, the early detection of vertebral osteomyelitis remains difficult. The onset of the symptom is often insidious and could easily be underestimated (or ignored) by both patients and doctors. The most common symptom is back pain; it may be insidious in onset during the early stages of infection but typically worsen at the advanced stage. Neurologic deficit may not be present until later in the course of disease. Other constitutional symptoms, such as fever, weight loss, chills, and anorexy are non-specific.Timely diagnosis of pyogenic vertebral osteomylitis depends on its consideration in patients with back pain and fever, especially those who are elderly, diabetic or immunocompromised. Delay in diagnosis of results in more severe tissue destruction, spinal instability and worsening neurological deficit [15]. So early diagnosisof PVO, especially identification of etiologic microorganism, become very important in the treatment of patients with PVO. The procedures for this step include blood cultures, percutaneous tissue biopsy and culture, and open biopsy and culture. When clinical history, laboratory values, and radiographic studies suggest spinal infection, blood cultures are firstly performed but show lower positive rates than other procedures [19]. When the result of blood cultures is negative, the procedures for obtaining infected tissue should be considered before antibiotic treatment starts [20]. In order to get enough tissue more accurately from the pathologic area, percutaneous CT-guided needle biopsy is a good option to enhance the culture result [21]. The low positive rate of percutaneous needle biopsy in our study may be due to failure in the procedure, low-virulence organisms, and prior antibiotic use before admission. If biopsy cultures fail to show an organism, histological analysis can often confirm the diagnosis [22].

Surgical treatment of PVO consists of radical debridement, reconstruction of anterior column with or without posterior stabilization aiming for fast postoperative mobilization [16]. There is a broad range of options for the surgical management of spinal infections, which include anterior or posterior approach, combined anteriror and posterior surgery, with or without instrumentation.

Because the inflammation is usually anterior to the neural contents, anterior operative approach is usually preferred. An anterior approach allows radical debridement, direct decompression and reconstruction of anterior column. However, this procedure has not been successful in preventing the progression of kyphosis or correcting the pre-existing kyphosis. And surgery performed with an anterior approach could result in higher mortality, especially for patients with combined cardiovascular and respiratory disease [23, 24]. Although anterior-only surgery can be performed, combined anteroposterior surgery provides for a more rigid spinal construct and better kyphosis correction. This combined procedure has a longer operation time, longer healing duration, and higher surgical trauma, especially for aged patients. The decision to perform anteroposterior spinal surgeries in a single-stage or 2-stage fashion is complex and remains controversial.

Nowadays, authors have tended to emphasize the importance of tailoring the management options according to patient general medical condition, degree of bony destruction and location of compressive lesions. Also, as posterior instrumentation has become popular as a technique to stabilize the unstable spine and more effective regimens of appropriate antibiotics have become available, posterior-only procedure become an alternative treatment of spinal infection [25]. We have treated contiguous spinal tuberculosis by posterior-only approach surgery and achieved good clinical effects [7–9]. We preferred posterior approach because some patients were in a poor medical condition and it was felt that surgery involving abdominal cavity would subject them to a high anaesthetic risk, with potential severe postoperative anterior complications. This approach far away from the abdominal cavity is characterized as the simple approach. It avoids high anaesthetic risk of anterior procedure with possibility to develop postoperative severe complications. Because there is no advantage of radical surgery over debridement when an extensive spinal lesion is present [26], we only removed focal tissues and tissues in focal edges, especially the sclerotic walls, caves, dead spaces, and so on, which could result in a incurative or recurrent result for vertebral osteomyelitis and reach the subnormal substance of bones between normal cancellous bones and pathologic bones. In our study, neurological function in patients with paraplegia was significantly improved postoperatively, which was similar to the result after anterior decompression [27].

The choice of the fixation range in lumbar segment remains controversial. Short-segment fixation provided stronger fixation and is still used by most surgeons for treatment of mono-segment lumbar PVO. Longer fixed segment range can distribute the longitudinal stress of the spine, which can significantly maintain the spinal stability and prevent loss of the correction. But short-segment fixation sacrificed two normal motion segments, affecting the activity of the lumbar spine in the long term and leading to aggravation of adjacent segment degeneration (ASD), which may ultimately cause ASD-related complications [28]. However, despite sacrificing more spinal functional units, our study have already demonstrated that compared with single-segment fixation, short-segment fixation can get better correction of kyphosis.

Recently, many studies reported that titanium mesh cages have been shown to be effective for reconstructing a deficient anterior column after a corpectomy in the treatment of PVO [4, 5, 29], by providing immediate spinal stability and improving sagittal balance, and thus facilitating bone healing. The use of titanium mesh cages has several benefits over other bone struts. The cage provides immediate stability, is rigid, and can tolerate compression forces well. The significant interface strength between the cage and endplates prevents it from extrusion or displacement. Most of all, the titanium mesh cage is the ideal shape, or it can be tailored to be positioned between adjacent vertebral endplates. It has relatively large weight-bearing surfaces. It is mechanically strong enough that can prevent from leading to discrete loss of height of a fused motion segment, which may be due to osteoporosis of the vertebrae [30]. Compression on the interbody titanium mesh cages allowed correction of the kyphosis.

Indications for the single-segment fixation techniques for the treatment of mono-segmental lumbar or lumbosacral PVO include: (1) lesions involved only a single motion segment, (2) ability to implant devices between adjacent vertebrae, (3) kyphosis angle < 20°. Contraindications to single-segment fixation include: (1) patients with severe kyphosis deformity, (2) presence of osteoporosis, (3) patients with vertebral osteomyelitis with bone healing or bone silent oscillation, accompanied by kyphosis deformity; these patients require osteotomy or internal fixation.

The limitations of our study include the retrospective nature of the report, the relatively small sample of patients in different groups. A much larger, randomized controlled trial is required to elucidate the benefits and risks of our method. However, the data here may serve as preliminary results that can aid surgeons and patients in decision-making and in the design of future well-designed prospective studies.

## Conclusions

Posterior-only debridement, interbody graft using titanium mesh cage, posterior single-segment instrumentation and fusion represents a safe and effective treatment option for selected patients with mono-segmental lumbar and lumbosacral PVO. This approach may preserve more lumbar normal motor units with less blood loss and operation time when compared with that of short-segment fixation. But short-segment fixation was superior to the single-segment fixation in the correction of kyphosis.

## Data Availability

The datasets and materials generated or analyzed during the current study are available from the corresponding author on reasonable request.

## Abbreviations

PVO: Pyogenic vertebral osteomyelitis
VAS: Visual analog scale
ESR: Erythrocyte sedimentation rate
CRP: C-reactive protein
ASIA: American Spinal Injury Association
CT: Computed tomography
MRI: Magnetic resonance imaging
COPD: Chronic obstructive pulmonary disease
ASD: Adjacent segment degeneration

## References

[1] Hidalgo-Ovejero AM, Otermin I, García-Mata S (1998). Pyogenic vertebral osteomyelitis. J Bone Jt Surg Am.

[2] An HS, Seldomridge JA (2006). Spinal infections: diagnostic tests and imaging studies. Clin Orthop Relat Res.

[3] Arnold PM, Baek PN, Bernardi RJ, Luck EA, Larson SJ (1997). Surgical management of nontuberculous thoracic and lumbar vertebral osteomyelitis: report of 33 cases. Surg Neurol.

[4] Kuklo RT, Potter BK, Bell SB, Moquin RR, Rosner MK (2006). Single-Stage treatment of pyogenic spinal infection with titanium mesh cages. J Spinal Disord Tech.

[5] Ruf M, Stoltze D, Merk RH, Ames M, Harms J (2007). Treatment of vertebral osteomyelitis by radical debridement and stabilization by radical debridement and stabilization using titanium mesh cages. Spine.

[6] Chiriano J, Abou-Zamzam AM, Urayeneza O, Zhang WW, Cheng W (2009). The role of the vascular surgeon in anterior retroperitoneal spine exposure: preservation of open surgical training. J Vasc Surg.

[7] Wang YX, Zhang HQ, Tang MX, Guo CF, Deng A, Wu JH, Liu JY, Deng ZJ, Chen J. One-stage posterior focus debridement, interbody grafts, and posterior instrumentation and fusion in the surgical treatment of thoracolumbar spinal tuberculosis with kyphosis in children: a preliminary report. Childs Nerv Syst. 2016; 32(8):1495–502. 10.1007/s00381-016-3152-9 27392447

[8] Wang YX, Zhang HQ, Li M, Tang MX, Guo CF, Deng A, Qile G, Jianhuang W, Jinyang L. Debridement, interbody graft using titanium mesh cages, posterior instrumentation and fusion in the surgical treatment of multilevel noncontiguous spinal tuberculosis in elderly patients via a posterior-only. Injury 2017;48(2):378–383. 10.1016/j.injury.2016.12.025 28063678

[9] Wang Y-X, Zhang H-q, Liao W, Tang M-X, Guo C-F, Deng A, Jianhuang Wu, Liu J (2016). One-stage posterior focus debridement, interbody graft using titanium mesh cages, posterior instrumentation and fusion in the surgical treatment of lumbo-sacral spinal tuberculosis in the aged. Int Orthop.

[10] Defino HL, Scarparo P (2005). Fractures of thoracolumbar spine: monosegmental fixation. Injury.

[11] Defino HL, Herrero CF, Romeiro CF (2007). Monosegmental fixation for the treatment of fractures of the thoracolumbar spine. Indian J Orthop.

[12] Zander T, Rohlmann A, Klockner C, Bergmann G. Comparison of the mechanical behavior of the lumbar spine following mono- and bisegmental stabilization. Clin Biomech. 2002;17(6):439–45. 10.1016/s0268-0033(02)00040-2 12135545

[13] Carragee EJ. Pyogenic vertebral osteomyelitis. J Bone J Surg Am 1997;79(6):874–880. 10.2106/00004623-199706000-00011 9199385

[14] Kehrer M, Pedersen C, Jensen TG, Lassen AT (2014). Increasing incidence of pyogenic spondylodiscitis: a 14-year population-based study. J Infect.

[15] Lora-Tamayo J, Euba G, Narváez JA, Murillo O, Verdaguer R, Sobrino B, Narváez J, Nolla JM, Ariza J. Changing trends in the epidemiology of pyogenic vertebral osteomyelitis: the impact of cases with no microbiologic diagnosis. Semin Arthritis Rheum. 2011;41(2):247–255. 10.1016/j.semarthrit.2011.04.002 21665246

[16] Chen WH, Jiang LS, Dai LY (2007). Surgical treatment of pyogenic vertebral osteomyelitis with spinal instrumentation. Eur Spine J.

[17] Belzunegui J, Intxausti JJ, De Dios JR, Del Val N, Rodriguez Valverde V, Gonzalez C, Queiro R, Figueroa M (2000). Haematogenous vertebral osteomyelitis in the elderly. Clin Rheumatol.

[18] Faraj AA, Webb JK (2000). Spinal instrumentation for primary pyogenic infection report of 31 patients. Acta Orthop Belg.

[19] Jeong SJ, Choi SW, Youm JY, Kim HW, Ha HG, Yi JS. Microbiology and epidemiology of infectious spinal disease. J Korean Neurosurg Soc. 2014;56(1):21–7. 10.3340/jkns.2014.56.1.21 PMC4185315 25289121

[20] Cottle L, Riordan T (2008). Infectious spondylodiscitis. J Infect.

[21] Gasbarrini A, Boriani L, Salvadori C, Mobarec S, Kreshak J, Nanni C, Zamparini E, Alberghini M, Viale P, Albisinni U (2012). Biopsy for suspected spondylodiscitis. Eur Rev Med Pharmacol Sci.

[22] Desoutter S, Cottier JP, Ghout I, Issartel B, Dinh A, Martin A, Carlier R, Bernard L; Duration of Treatment for Spondylodiscitis Study Group.Duration of Treatment for Spondylodiscitis Study Group. Susceptibility pattern of microorganisms isolated by percutaneous needle biopsy in nonbacteremic pyogenic vertebral osteomyelitis. Antimicrob Agents Chemother. 2015;59(12):7700–6. 10.1128/AAC.01516-15 PMC4649186 26438497

[23] Zhong-Quan F, Zhong-Min Z, Da-Di J, Jian-Ting C, Dong-Bin Q. Complications of the anterior surgical approach for thoracolumbar spine tuberculosis: causes and countermeasures. Nan Fang Yi Ke Da Xue Xue Bao. 2009;29(6):1229–31. 19726369

[24] Zhongyang L, Fei L, Peng X, Xi Y, Lei W, Ganjun F, Limin L, Yueming S, Chunguang Z. Surgical management for middle or lower thoracic spinal tuberculosis (T5-T12) in elderly patients: posterior versus anterior approach. J o Orthop Sci. 2018;110:e842–e850. 10.1016/j.jos.2018.08.012 30245090

[25] Gorensek M, Kosak R, Travnik L, Vengust R. Posterior instrumentation, anterior column reconstruction with single posterior approach for treatment of pyogenic osteomyelitis of thoracic and lumbar spine. Eur Spine J. 2013;22(3):633–41. 10.1007/s00586-012-2487-5 PMC3585646 22922802

[26] Jain AK (2002). Treatment of tuberculosis of the spine with neurologic complications. Clin Orthop Rel Res.

[27] Dai LY, Chen WH, Jiang LS (2008). Anterior instrumentation for the treatment of pyogenic vertebral osteomyelitis of thoracic and lumbar spine. Eur Spine J.

[28] Maruenda JI, Barrios C, Garibo F, Maruenda B (2016). Adjacent segment degeneration and revision surgery after circumferential lumbar fusion: outcomes throughout 15 years of follow-up. Eur Spine J.

[29] Robertson PA, Rawlinson HJ, Hadlow AT (2004). Radiologic tability of titanium mesh cages for anterior spinal reconstruction following thoracolumbar corpectomy. J Spinal Disord Tech.

[30] Pee YH, Park JD, Choi YG, Lee SH (2008). Anterior debridement and fusion followed by posterior pedicle screw fixation in pyogenic spondylodiscitis: autologous iliac bone strut versus cage. J Neurosurg Spine.

